# IL-7/IL-7R在非小细胞肺癌中的表达及与淋巴转移和预后的关系

**DOI:** 10.3779/j.issn.1009-3419.2010.12.04

**Published:** 2010-12-20

**Authors:** 健 明, 清富 张, 彦多 姜, 雪杉 邱, 晓忠 白

**Affiliations:** 1 110003 沈阳，解放军第二零二医院病理科 No.202 Hospital of People Liberation Army of China, 110003 Shenyang, China; 2 110001 沈阳，中国医科大学附属第一医院病理科，中国医科大学病理教研室 Department of Pathology, College of Basic Medical Sciences, China Medical University and Department of Pathology, the First Affiliated Hospital of China Medical University, Shenyang 110001, China

**Keywords:** 肺肿瘤, 白介素7, 血管内皮生长因子-D, 转移, Lung neoplasms, Interleukin-7, Vascular endothelial growth factor-D, Metastasis

## Abstract

**背景与目的:**

已有的研究表明淋巴转移与肺癌的预后密切相关，白介素-7（interleukin-7, IL-7）和IL-7受体（interleukin-7 receptor, IL-7R）可以通过血管内皮生长因子-D（vascular endothelial growth factor-D, VEGF-D）促进淋巴转移。本研究旨在探讨IL-7和IL-7R在非小细胞肺癌（non-small cell lung cancer, NSCLC）中的表达情况，分析它们与各临床病理因素、VEGF-D及预后之间的关系。

**方法:**

免疫组化方法检测95例原发性NSCLC组织标本中IL-7和IL- 7R的表达情况，分析它们与各临床病理因素、VEGF-D及预后之间的关系。

**结果:**

95例原发性NSCLC组织中IL-7、IL-7R和VEGF-D高表达者分别占63.16%、61.05%和58.95%，IL-7和IL-7R的表达与临床分期和淋巴结转移均密切相关，而与患者的年龄、性别、组织分型、分化程度无明显关系；IL-7和IL-7R与VEGF-D高表达组的淋巴管密度（lymphatic vessel density, LVD）明显高于低表达或无表达组的LVD，差异具有统计学意义（*P*=0.003，*P*=0.019和*P* < 0.001）；生存分析显示IL-7和IL-7R与VEGF-D高表达组的预后较差。

**结论:**

在NSCLC中IL-7/IL-7R高表达与分期、淋巴结转移、VEGF-D、LVD和预后不良呈正相关。

## Abstract (en)

**Background and objective:**

It has been proven that lymph node metastasis was closely related to prognosis of lung cancer. Interleukin-7 (IL-7) and interleukin-7 receptor (IL-7R) could promote lymph node metastasis through vascular endothelial growth factor-D (VEGF-D). The aim of this study is to explore the expressions of IL-7 and IL-7R in lung cancer and the relationship between them with lymph node metastasis and prognosis in non-small cell lung cancer (NSCLC).

**Methods:**

The expressions of IL-7 and IL-7R in 95 cases of NSCLC were detected with immunohistochemistry method and the relationship between IL-7 and IL-7R and their impact on lung cancer patients' outcomes were analyzed.

**Results:**

In 95 cases of NSCLC, the high expression rates of IL-7, IL-7R and VEGF-D were 63.16%, 61.05% and 58.95%. The expressions of IL-7 and IL-7R were correlated closely with clinic stage and lymph node metastasis, but had no relationship with age, gender, histological type and differentiation degree. The lymphatic vessel density (LVD) mean of the group with high expressions of IL-7 and IL-7R was higher than that with low or negative expressions of IL-7 and IL-7R, and they were significant different in statistics. *Log-rank* analysis showed that the postoperative survival period was significantly shorter in high expression groups IL-7, IL-7R and VEGF-D comparing with that in low or negative groups.

**Conclusion:**

The high expression of IL-7 and IL-7R is highly positie correlated with clinic stage, lymph node metastasis, VEGF-D, LVD and poor prognosis in Non-small cell lung cancer.

白介素-7（interleukin-7, IL-7）是一种细胞因子，它主要由胸腺细胞^[1, 2]^、骨髓基质细胞、小肠上皮细胞^[3]^和皮肤角化细胞^[4]^等分泌，在人体免疫系统的正常发育和维持正常免疫功能中起重要作用。IL-7在维持人体免疫系统稳态中起重要作用。同时，它能诱导造血细胞和血液恶性肿瘤细胞（如：白血病和淋巴瘤）生长和增殖^[5-10]^。但是，关于它在实体肿瘤中的表达和作用的研究较少。IL-7在肺癌组织中如何表达及其表达与肺癌转移是否存在相关性尚未见报道。本实验将探讨非小细胞肺癌（non-small cell lung cancer, NSCLC）组织中IL-7、IL-7受体（interleukin-7 receptor, IL-7R）和血管内皮生长因子-D（vascular endothelial growth factor-D, VEGF-D）表达与临床病理因素、淋巴结转移和预后的关系。

## 1 材料与方法

### 1.1 材料

选取1980年1月-2001年12月在中国医科大学附属第一医院行肺癌根治性切除且有完整随访资料的NSCLC组织95例。同时取相应的癌旁肺组织做对照，所取癌旁肺组织距癌灶边缘5 cm以上，并且经HE染色证实。所有患者术前均未接受过任何放疗或化疗，男性71例，女性24例；年龄32岁-72岁（58.50岁±8.96岁）。按照国际抗癌联盟（International Union Against Cancer, UICC）1997年修订的肺癌pTNM分期标准，Ⅰ期33例，Ⅱ期9例，Ⅲ期53例；中高分化56例，低分化39例；淋巴结转移58例。所有标本的使用均征得患者本人同意。生存时间的计算是从手术日期到由于复发/转移而死亡的日期或末次随访日期为止，随访期1个月-117个月，平均23.84个月，随访中发现47例患者发生血行转移，其中脑转移19例、骨转移14例、肾上腺转移14例。

### 1.2 方法

95例原发性NSCLC癌组织及癌旁正常肺组织标本，用中性福尔马林溶液固定，石蜡包埋，制成4 μm切片，采用SP法检测组织中IL-7、IL -7R和VEGF-D蛋白表达情况以及微血管密度（microvessel density, MVD）和淋巴管密度（lymphatic vessel density, LVD）。高温高压抗原修复，一抗IL-7（1:100）、IL-7R（1:100）、VEGF-D（1:150）抗体均购自美国Santa Cruz，D2-40抗体和CD34抗体分别购自DAKO和美国Lab Vision。SP试剂盒和二氨基联苯胺（diaminobenzidine, DAB）酶底物显色试剂盒（DAB-0031）购自迈新公司。用PBS代替一抗作为阴性对照。结果判定：IL-7、IL-7R和VEGF-D以细胞质中出现棕黄色颗粒为阳性显色。光镜下每张切片选取癌细胞较多的10个高倍视野计数100个癌细胞中的阳性细胞数，IL-7、IL-7R和VEGF-D的评估为：无阳性细胞为“-”，阳性细胞数≤5%为“+”，阳性细胞数占6%-20%为“++”，阳性细胞数 > 20%为“+++”，其中“++”和“+++”为高表达，“-”和“+”为低表达。血管和淋巴管判定标准参考Weinder^[11]^标准。血管和淋巴管判定标准为：内皮细胞形成条状、裂隙状等孤立结构棕黄染色或有管腔者按一条血管或淋巴管计数。低倍光镜下确定3个微血管或淋巴管高密度区域（热点），然后在高倍镜下分别计数每个热点中3个区域的CD34染色（用于标记MVD）和D2-40（用于标记LVD）染色阳性管腔数均值。MVD=（CD34阳性管腔数-D2-40阳性管腔数）均值；LVD=D2-40阳性管腔数均值。当计数相差10%以上时则重新计数。

### 1.3 统计分析

采用SPSS 13.0统计软件，采用*χ*^2^检验分析IL-7表达和临床病理因素的关系；生存分析采用*Kaplan- Meier*法，采用*Log-rank*检验判断差异性。*P* < 0.05为有统计学差异。

## 2 结果

### 2.1 IL-7/IL-7R的表达与NSCLC临床病理因素的关系

95例NSCLC组织免疫组织化学检测结果显示：在正常支气管上皮、腺体和肺泡上皮细胞未见IL-7和IL-7R的表达（图 1A，图 1C）。IL-7主要在癌细胞胞质内表达（图 1B），按评分标准，高表达者占63.16%（60/95），低表达者或无表达者占36.84%（35/95）；IL-7R主要在癌细胞胞质内和胞膜上表达（图 1D），高表达者占61.05%（58/95），低表达者或无表达者占38.95%（37/95）。IL-7的表达水平与IL-7R明显相关（*r*=47.906, *P* < 0.001）。

**1:**
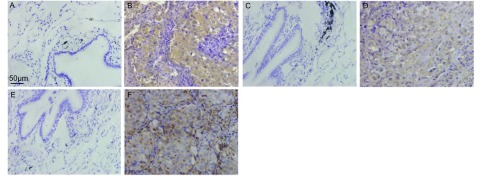
在正常肺组织中IL-7（A）、IL-7R（C）和VEGF-D（E）呈阴性表达，肺癌中IL-7（B）和VEGF-D（F）主要在肿瘤细胞质中表达，IL-7R主要在肿瘤细胞质和细胞膜上表达（D）（SP，×400)。 The expressions of IL-7 (A), IL-7R (C) and VEGF-D (E) is negative in normal lung tissue; the expressions of IL-7 (B) and VEGF-D (D) are positive in cytoplasm of lung cancer cells; the expressions of IL-7R is positive in cytoplasm and membrane of lung cancer cell (D) (SP, ×400).

对95例NSCLC组织中IL -7和IL -7R的表达情况与临床病理因素进行*χ*^2^检验、*t*检验等统计学分析。结果显示：IL-7和IL-7R的表达与临床分期（*P*=0.001; *P*=0.005）和淋巴结转移（*P* < 0.001; *P* < 0.001）均密切相关，而与患者的年龄、性别、组织分型、分化程度无明显关系（*P* > 0.05）（表 1）。

**1:** IL-7和IL-7R蛋白表达与NSCLC患者临床因素之间的关系 Relationship between IL-7 and IL-7R expression in NSCLC and clinical pathological factors

Factors	*n*	IL-7 expression [*n* (%)]	*P*	IL-7R expression [*n* (%)]	*P*
Gender			0.571		0.752
Male	71	46（64.79%）		44（61.97%）	
Female	24	14（58.33%）		14（58.33%）	
Age (years)			0.356		0.169
≤60	52	35（67.31%）		35（67.31%）	
> 60	43	25（58.14%）		23（53.49%）	
Histology			0.664		0.606
Squamous cance	57	35（61.40%）		36（63.16%）	
Adenocarcinoma	38	25（65.79%）		22（57.89%）	
Differentiation			0.255		0.439
Well & Moderate	56	38（67.86%）		36（64.29%）	
Poor	39	22（56.41%）		22（56.41%）	
Lymph node metas			< 0.001^*^		< 0.001^*^
Positive	58	45（77.59%）		44（75.86%）	
Negative	37	15（40.54%）		14（37.84%）	
Distant metastasis			0.188		0.326
Positive	47	31（65.96%）		32（68.09%）	
Negative	48	23（47.92%）		26（54.17%）	
Stage			0.001^*^		0.005^*^
Ⅰ-Ⅱ	42	19（42.86%）		19（42.86%）	
Ⅲ-Ⅳ	53	41（77.36%）		39（73.58%）	
^*^P < 0.05; NSCLC: non-small cell lung cancer; IL-7: interleukin-7; IL-7R: interleukin-7 receptor.					

### 2.2 IL-7和IL-7R表达与VEGF-D表达的关系

95例人NSCLC免疫组织化学检测结果显示：在正常支气管上皮、腺体和肺泡上皮细胞均未见到VEGF-D的表达（图 1E），在间质的血管、淋巴管内皮细胞内有VEGF-D的表达。VEGF-D主要表达于癌细胞胞质内（图 1F），按评分标准，高表达者占58.95%（56/95），低表达者或无表达者占41.05%（39/95）。IL -7的表达水平与VEGF-D表达水平呈正相关（*r*=19.189, *P* < 0.001）；IL-7R的表达水平与VEGF-D表达水平呈正相关（*r*=9.777, *P*=0.001）。

### 2.3 IL-7、IL-7R和VEGF-D的表达与MVD和LVD的关系

我们分别计数IL -7、IL -7R和VEGF-D高表达组和低表达或无表达组的血管和淋巴管数目。肿瘤内的血管和淋巴管分别用CD34和D2-40抗体标记，呈条索或裂隙状。IL -7高表达组的LVD明显高于低表达或无表达组，差异具有统计学意义（*P*=0.003）；IL-7R高表达组的LVD明显高于低表达或无表达组，差异具有统计学意义（*P*=0.019）；VEGF-D高表达组的LVD明显高于低表达或无表达组，差异具有统计学意义（*P* < 0.001）（表 2）。结果显示IL-7、IL-7R和VEGF-D的表达与NSCLC组织的LVD有关，而与MVD无关联（图 2）。

**2:** 在NSCLC中IL-7、IL-7R和VEGF-D表达与MVD、LVD的关系 Relationship among the expressions of IL-7, IL-7R and VEGF-D with MVD and LVD in NSCLC

	*n*	MVD	*P*	LVD	*P*
IL-7			0.435		0.003
High expression	60	41.29±18.24		30.19±14.60	
Low or no expression	35	38.56±15.22		21.36±11.52	
IL-7R			0.334		0.019
High expression	58	41.60±18.55		29.65±14.98	
Low or no expression	37	38.26±14.81		22.69±11.70	
VEGF-D			0.378		< 0.001
High expression	56	40.80±13.54		34.11±12.11	
Low or no expression	39	37.78±19.76		16.64±9.91	
VEGF-D: vascular endothelial growth factor-D; MVD: microvessel density; LVD: lymphatic vessel density.					

**2:**
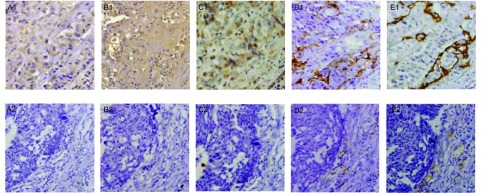
NSCLC中IL-7、IL-7R、VEGF-D的表达与MVD和LVD之间的关系。当IL-7（A1）、IL-7R（B1）、VEGF-D（C1）呈高表达时相应的LVD（E1）也呈高表达；当IL-7（A2）、IL-7R（B2）、VEGF-D（C2）呈阴性表达时相应的LVD（E2）也呈低表达，而MVD（D1、D2）与三者无关（SP，×400）。 Relationship of the expressions of IL-7, IL-7R, and VEGF-D in NSCLC with MVD and LVD. When the expression levels of IL-7 (A1), IL-7R (B1) and VEGF-D (C1) were high, the corresponding LVD was also high (E1); However, when the expression levels of IL-7 (A2), IL-7R (B2), and VEGF-D (C2) were low, the corresponding LVD were significantly low (E2), but MVD was not related with the expressions of IL-7, IL-7R, and VEGF-D (SP, ×400).

### 2.4 IL-7、IL-7R和VEGF-D表达与NSCLC预后的关系

对95例有完整的术后随访记录的患者进行*Kaplan-Meier*生存曲线分析显示：IL-7高表达组患的生存时间明显低于低表达或无表达组者（16±4.487 *vs* 24±4.982, *P*=0.008）；IL-7R高表达组患者的5年生存率明显低于低表达或无表达组（18±4.152 *vs* 22±3.649, *P*=0.032）；VEGF-D高表达组患者的5年生存率明显低于低表达或无表达组（16±3.813 *vs* 23 ±1.369, *P*=0.028）（图 3）。

**3:**
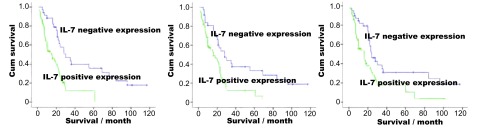
IL-7（A）、IL-7R（B）和VEGF-D（C）阴性表达组和阳性表达组患者的*Kaplan-Meier*生存曲线（*Log-rank*检验，*P*=0.008，*P*=0.032，*P*=0.028）。 Survival of NSCLC patients correlates with the expression of IL-7, IL-7R and VEGF-D. *Kaplan-Meier* survival plots for patients with NSCLC, grouped according to IL-7 (A), IL-7R (B) and VEGF-D (C) protein expression (*Log-rank* test, *P*=0.008, *P*=0.032, *P*=0.028).

## 3 讨论

近年研究显示，一些恶性肿瘤，如：慢性淋巴细胞白血病细胞^[8]^、Burkitt’s淋巴瘤细胞^[9]^和结肠癌细胞^[10, 12]^能产生IL-7；还有一些肿瘤细胞系，如：食道癌^[13]^、肾癌^[14]^、头颈鳞状细胞癌^[15]^和腮腺Warthin’s细胞系^[16]^表达*IL-7*基因。另外，Al-Rawi^[17]^研究了乳腺癌组织中IL-7和IL-7R的表达，结果显示在乳腺癌组织中IL-7阴性表达，而IL-7R表达阳性，且IL-7R表达与肿瘤淋巴结转移有关。

IL-7在肺癌组织中表达如何及其表达与肺癌转移是否存在相关性尚未见报道。为了探讨IL-7在肺癌组织中的表达情况，本研究通过免疫组化的方法检测其在95例有完整随访资料的NSCLC以及癌旁组织的表达，结果发现肺癌组织中IL-7呈高表达，在正常支气管上皮、腺体和肺泡上皮细胞中不表达。Al-Rawi^[17]^研究发现IL-7在乳腺癌组织中不表达，这与我们的研究结果不一致，这可能是组织特异性造成的，而在乳腺癌组织中有IL-7R的表达，IL -7R的表达与肿瘤转移到局部淋巴结有关，这可能是血液中或其它细胞分泌的IL-7作用于IL-7R受体后引起的相关作用。我们发现在肺癌中IL-7和IL-7R均呈高表达，且与分期和淋巴结转移呈正相关。

随着肿瘤淋巴管形成相关分子血管内皮生长因子-C（vascular endothelial growth factor-C, VEGF-C）和VEGF-D被发现，肿瘤淋巴管形成成为人们关注的热点。在动物模型中，VEGF-C和VEGF-D可以诱导肿瘤淋巴管形成^[18, 19]^。在人膀胱癌、甲状腺乳头癌、前列腺癌、胃癌、结直肠癌、乳腺癌、卵巢癌和肺癌^[20-28]^中VEGF-D高表达与肿瘤淋巴结转移密切相关。另外，免疫组化结果显示在许多肿瘤细胞中VEGF-D呈高表达，这说明肿瘤细胞自身可以产生VEGF-D，促进肿瘤淋巴管形成^[29]^。

淋巴转移和血行转移是肺癌主要转移途径，新生血管和淋巴管的形成是肿瘤血行转移和淋巴转移的基础，VEGF-D属于VEGF家族，与其受体VEGFR-3结合后可引起淋巴管内皮细胞增生，促进淋巴管形成，VEGF-D在肿瘤淋巴管形成过程中发挥重要作用。我们同时检测了肿瘤组织周围MVD和LVD，发现在肺癌中，淋巴转移和血行转移组肿瘤组织周围LVD和MVD明显高于非转移组，且IL-7和IL-7R高表达组肿瘤中的LVD明显高于低表达或无表达组，而MVD无明显差异。

为了进一步解释I L -7高表达时LVD数目增多的现象，我们又检测了这些组织中VEGF-D的表达，结果发现VEGF-D与LVD呈正相关，且IL -7和IL -7R高表达均与VEGF-D的阳性表达呈正相关。Al-Rawi^[30, 31]^研究发现IL-7可以促进乳腺癌细胞系中VEGF-D的表达，同时促进淋巴管形成。因此我们认为在肺癌中IL -7/IL -7R高表达促进VEGF-D高表达，从而促进淋巴管形成。

对所有具有临床随访资料的患者进行生存曲线分析发现，IL-7和IL-7R低表达或无表达组的患者的5年生存率明显高于高表达组的患者，表明IL-7和IL-7R表达的上调可降低患者的生存时间和生存率。

综上，在NSCLC组织中，IL-7和IL-7R的表达水平与VEGF-D表达、肿瘤分期、淋巴结转移、LVD和预后不良呈正相关。

## References

[1] Namen AE, Lupton S, Hjerrild K (1988). Stimulation of B-cell progenitors by cloned murine interleukin-7. Nature.

[2] Wiles MV, Ruiz P, Imhof BA (1992). Interleukin-7 expression during mouse thymus development. Eur J Immunol.

[3] Watanabe M, Ueno Y, Yajima T (1995). Interleukin-7 is produced by human intestinal epithelial-cells and regulates the proliferation of intestinal mucosal lymphocytes. J Clin Invest.

[4] Heufler C, Topar G, Grasseger A (1993). Interleukin 7 is produced by murine and human keratinocytes. J ExpMed.

[5] Touw I, Pouwels K, van Agthoven T (1990). Interleukin-7 is a growth factor of precursor B and T acute lymphoblastic leukemia. Blood.

[6] Qin JZ, Zhang CL, Kamarashev J (2001). Interleukin-7 and interleukin-15 regulate the expression of the *bcl*-2 and *c-myb* genes in cutaneous T-cell lymphoma cells. Blood.

[7] Foss HD, Hummel M, Gottstein S (1995). Frequent expression of *IL*-7 gene transcripts in tumor cells of classical Hodgkin's disease. Am J Pathol.

[8] Frishman J, Long B, Knospe W (1993). Genes for interleukin-7 are transcribed in leukemic-cell subsets of individuals with chronic lymphocytic-leukemia. J Exp Med.

[9] Takakuwa T, Nomura S, Matsuzuka F (2000). Expression of interleukin-7 and its receptor in thyroid lymphoma. Lab Invest.

[10] Yamada K, Shimaoka M, Nagayama K (1997). Bacterial invasion induces interleukin- 7 receptor expression in colonic epithelial cell line, T84. Eur J Immunol.

[11] Weidner N (1995). Current pathologic methods for measuring intratumoral microvessel density within breast carcinoma and other solid tumors. Breast Cancer Res Treat.

[12] Maeurer MJ, Walter W, Martin D (1997). Interleukin-7 (IL-7) in colorectal cancer: IL-7 is produced by tissues from colorectal cancer and promotes preferential expansion of tumour infiltrating lymphocytes. Scand J Immunol.

[13] Oka M, Hirose K, Iizuka N (1995). Cytokine mRNA expression patterns in human esophageal cancer cell lines. J Interferon Cytokine Res.

[14] Trinder P, Seitzer U, Gerdes J (1999). Constitutive and IFN-gamma regulated expression of IL-7 and IL-15 in human renal cell cancer. Int J Oncol.

[15] Paleri V, Pulimood A, Davies GR (2001). Interleukins 7 and 12 are expressed in head and neck squamous cancer. Clin Otolaryngol.

[16] Takeuchi T, Yamanouchi H, Yue Q (1998). Epithelial component of lymphoid stroma-rich Warthin's tumour expresses interleukin (IL)-7. Histopathology.

[17] Al-Rawi M, Mansel R, Jiang W (2002). Interleukin-7 (IL-7) and IL-7 receptor expression in breast cancer. Breast Cancer Res Treat.

[18] Stacker SA, Caesar C, Baldwin ME (2001). VEGF-D promotes the metastatic spread of tumor cells via the lymphatics. Nat Med.

[19] Skobe M, Hawighorst T, Jackson DG (2001). Induction of tumor lymphangiogenesis by VEGF-C promotes breast cancer metastasis. Nat Med.

[20] Makinen T, Veikkola T, Mustjoki S (2001). Isolated lymphatic endothelial cells transduce growth, survival and migratory signals via the VEGF-C/D receptor VEGFR-3. EMBO J.

[21] Herrmann E, Eltze E, Bierer S (2007). VEGF-C, VEGF-D and Flt-4 in transitional bladder cancer: relationships to clinicopathological parameters and long-term survival. Anticancer Res.

[22] Maekawa S, Iwasaki A, Shirakusa T (2007). Correlation between lymph node metastasis and the expression of VEGF-C, VEGF-D and VEGFR-3 in T1 lung adenocarcinoma. Anticancer Res.

[23] van Iterson V, Leidenius M, von Smitten K (2007). VEGF-D in association with VEGFR-3 promotes nodal metastasis in human invasive lobular breast cancer. Am J Clin Pathol.

[24] Yonemura Y, Endo Y, Tabata K (2005). Role of VEGF-C and VEGF-D in lymphangiogenesis in gastric cancer. Int J Clin Oncol.

[25] Stearns ME, Wang M, Hu Y (2004). Expression of a flt-4 (VEGFR3) splicing variant in primary human prostate tumors. VEGF D and flt-4t (Delta773- 1081) overexpression is diagnostic for sentinel lymph node metastasis. Lab Invest.

[26] Yasuoka H, Nakamura Y, Zuo H (2005). VEGF-D expression and lymph vessels play an important role for lymph node metastasis in papillary thyroid carcinoma. Mod Pathol.

[27] Onogawa S, Kitadai Y, Tanaka S (2004). Expression of VEGF-C and VEGF-D at the invasive edge correlates with lymph node metastasis and prognosis of patients with colorectal carcinoma. Cancer Sci.

[28] Rossochacka-Rostalska B, Gisterek I, Suder E (2006). Vascular endothelial growth factor-C (VEGF-C) and vascular endothelial growth factor-D (VEGF-D) in ovarian carcinomas. Ginekol Pol.

[29] Stacker SA, Baldwin ME, Achen MG (2002). The role of tumor lymphangiogenesis in metastatic spread. FASEB J.

[30] Al-Rawi MA, Watkins G, Mansel RE (2005). Interleukin 7 upregulates vascular endothelial growth factor D in breast cancer cells and induces lymphangiogenesis *in vivo*. Br J Surg.

[31] Al-Rawi MA, Watkins G, Mansel RE (2005). The effects of interleukin-7 on the lymphangiogenic properties of human endothelial cells. Int J Oncol.

